# Inflammatory CSF profiles and longitudinal development of cognitive decline in sporadic and GBA-associated PD

**DOI:** 10.1038/s41531-023-00476-2

**Published:** 2023-03-11

**Authors:** Stefanie Lerche, Milan Zimmermann, Benjamin Roeben, Isabel Wurster, Franca Laura Fries, Christian Deuschle, Katharina Waniek, Ingolf Lachmann, Meike Jakobi, Thomas O. Joos, Thomas Knorpp, Nicole Schneiderhan-Marra, Kathrin Brockmann

**Affiliations:** 1 Center of Neurology, Department of Neurodegeneration and Hertie-Institute for Clinical Brain Research, University of Tuebingen, Tuebingen, Germany; 2 German Center for Neurodegenerative Diseases, University of Tuebingen, Tuebingen, Germany; 3 Roboscreen GmbH, Leipzig, Germany; 4 Natural and Medical Sciences Institute at the University of Tübingen (NMI), Reutlingen, Germany

**Keywords:** Parkinson's disease, Neuroimmunology, Cognitive ageing, Predictive markers

## Abstract

Inflammation modifies the incidence and progression of Parkinson’s disease (PD). By using 30 inflammatory markers in CSF in 498 people with PD and 67 people with dementia with Lewy bodies (DLB) we show that: (1) levels of ICAM-1, Interleukin-8, MCP-1, MIP-1 beta, SCF and VEGF were associated with clinical scores and neurodegenerative CSF biomarkers (Aβ1-42, t-Tau, p181-Tau, NFL and α-synuclein). (2) PD patients with GBA mutations show similar levels of inflammatory markers compared to PD patients without GBA mutations, even when stratified by mutation severity. (3) PD patients who longitudinally developed cognitive impairment during the study had higher levels of TNF-alpha at baseline compared to patients without the development of cognitive impairment. (4) Higher levels of VEGF and MIP-1 beta were associated with a longer duration until the development of cognitive impairment. We conclude that the majority of inflammatory markers is limited in robustly predicting longitudinal trajectories of developing cognitive impairment.

## Introduction

Parkinson’s disease (PD) is a multifactorial disorder with age, genetics, environmental and life-style factors contributing to disease manifestation and clinical trajectories. In recent years, a growing number of epidemiological and genetic studies as well as post-mortem, biofluid and cell model analyses provided robust evidence for an additional influence of inflammation on incidence and progression in PD^1–6^. In this context, the cerebral and peripheral, as well as the innate and adaptive immune systems seem involved^7^. The activation of microglia as representative of the cerebral innate immune system was shown post-mortem and in vivo in people with PD by positron emission tomography studies and by increased levels of cytokines in cerebrospinal fluid (CSF)^2,8^. Microglia activation is driven by damage-associated molecular patterns (DAMPs), which originate from damaged cells, misfolded proteins, and protein aggregates. In PD, misfolded α-synuclein acts as DAMP resulting in NLRP3 (NOD-, LRR- and pyrin domain-containing protein 3) inflammasome and microglia activation with release of cytokines and induction of neuroinflammation^9,10^. Similar to findings in the brain, α-synuclein also promotes inflammasome-related cytokine production in the periphery. Moreover, specific α-synuclein peptides act as antigenic epitopes resulting in helper and cytotoxic T-cell responses in peripheral blood mononuclear cell (PBMC) from people with PD^11,12^.

Post-mortem and biofluid (blood, CSF) studies reported that increased inflammatory profiles are associated with clinical subtypes of PD, promoting an accelerated motor and non-motor phenotype^3,13–17^. Recent evidence highlights that the involvement of inflammation in PD is maximised in the early disease stages and maintains a chronic profile during the course of the disease^18,19^.

Importantly, correlations of inflammatory markers in matched blood and CSF pairs are sparse, indicating that they do not directly reflect one another^15,20,21^. This poses a challenge for clinical trials to decide which biomaterial should be collected for repeated outcome measurements.

Despite the clear role of inflammation in PD, the following shortcomings remain: (I) Prospective longitudinal studies assessing multiple inflammatory markers are sparse, specifically for CSF. (II) In order to enrich cohorts for maximised therapeutic effects in clinical trials targeting inflammation, knowledge of the predictive/prognostic value of inflammatory profiles in relation to clinical trajectories is important. (III) We lack comprehensive biofluids analyses assessing CSF levels of multiple inflammatory markers along with CSF levels of neurodegenerative/PD-specific biomarkers such as Amyloid-β_1-42_ (Aβ_1-42_), total-Tau (t-Tau), phospho-Tau (p181-Tau), neurofilament light chain (NFL), and α-synuclein. (IV) Studies in people with genetic forms of PD are just beginning to emerge, with no longitudinal reports so far. (V) Only a few studies stratified their analyses by sex. However, females and males differ in their immune systems as they show distinct patterns in innate and adaptive immune responses, which further change across life-span (22). In general, females show stronger innate and adaptive immune responses than males. Taking these gender differences into consideration is important when analysing immune marker profiles against disease status.

We aimed to shed some light on these points and assessed 30 inflammatory markers in CSF in a large longitudinal cohort of 498 people with PD, including 98 with mutations in the gene *glucocerebrosidase* (*GBA*) as a proxy for a more rapid cognitive decline in PD^22,23^. Moreover, 67 people diagnosed with dementia with Lewy bodies (DLB) have been included as a proxy of a clear endpoint (dementia) spanning the clinical and histopathological continuum as α-synucleinopathy between PD, PD with cognitive impairment and DLB. There are some data on inflammation in cohorts with *GBA* mutation carriers. While one small study reports increased plasma levels of IFN-γ, IL-1β, IL-2 and TNF-α in eight PD patients^24^, two larger studies did not find any differences in blood and CSF levels between PD patients and healthy individuals with vs. without heterozygous *GBA* mutations^25,26^.

## Results

### Participants

Between 2005 and 2020, the CSF of 498 PD patients (including 98 patients carrying a *GBA* mutation PD_GBA_: 48 with risk mutation = PD_GBA_risk_, 20 with mild mutation = PD_GBA_mild_, 30 with severe mutation = PD_GBA_severe_) and 67 DLB patients (including 19 patients carrying a GBA mutation: risk mutation *n* = 12, mild mutation *n* = 2, severe mutation *n* = 5) recruited from the outpatient clinic and/or ward for PD at the University Hospital of Tübingen were collected.

Male PD patients presented with a mean age of 65 years, mean age at onset of 58 years, mean disease duration of 7 years, mean H&Y of 2.1, mean UPDRS-III of 27, mean MoCA of 25 and mean LEDD of 600 mg. Mean CSF levels of neurodegenerative and PD-associated markers in pg/ml were as follows: Aβ_1-42_: 712, t-Tau: 230, p181-Tau: 40, NFL: 1042, total α-synuclein: 569. Female PD patients presented with a mean age of 66 years, mean age at onset of 59 years, mean disease duration of 7 years, mean H&Y of 2.1, mean UPDRS-III of 25, mean MoCA of 25 and mean LEDD of 533 mg. Mean CSF levels of neurodegenerative and PD-associated markers in pg/ml were as follows: Aβ_1-42_: 705, t-Tau: 267, p181-Tau: 44, NFL: 927, total α-synuclein: 679. Only eight patients (1.6 %) reported a daily intake of Ibuprofen.

Male DLB patients presented with a mean age of 71 years, mean age at onset of 67 years, mean disease duration of 5 years, mean H&Y of 2.3, mean UPDRS-III of 25, mean MoCA of 14, and mean LEDD of 366 mg. Mean CSF levels of neurodegenerative and PD-associated markers in pg/ml were as follows: Aβ_1-42_: 476, t-Tau: 294, p181-Tau: 44, NFL: 1613, total α-synuclein: 471. Female DLB patients presented with a mean age of 75 years, mean age at onset of 73 years, mean disease duration of 2 years, mean H&Y of 2.7, mean UPDRS-III of 24, mean MoCA of 13, and mean LEDD of 396. Mean CSF levels of neurodegenerative and PD-associated markers in pg/ml were as follows: Aβ_1-42_: 522, t-Tau: 327, p181-Tau: 47, NFL: 1785, total α-synuclein: 564.

## Cross-sectional

### Similar inflammatory CSF levels in PD patients with and without GBA mutations

Compared to PD_GBA_WT_, PD_GBA_ patients showed similar CSF levels of all assessed inflammatory markers except for TNF-alpha. Levels of TNF-alpha were lower in PD_GBA_ patients compared to PD_GBA_WT_ patients (3.14 pg/ml versus 3.72 pg/ml, *p* = 0.003). No gradual effects of *GBA* mutation severity were observed for any of the markers. Table 1.

**Table 1:** Demographic, clinical and CSF inflammatory characteristics in people with PD stratified by GBA mutation.

	PD_GBA_WT_ *n* = 400	PD_GBA_ *n* = 98	*p* value	PD_GBA_risk_ *n* = 48	PD_GBA_mild_ *n* = 20	PD_GBA_severe_ *n* = 30	*p* value^b^
Male sex, % (*n*)	64 (256)	67 (66)	0.558	73 (35)	60 (12)	63 (19)	0.632
Age	66 ± 10	63 ± 9	0.029	65 ± 9	64 ± 10	59 ± 9***##	0.007
Age at onset	59 ± 11	55 ± 10	<0.001	58 ± 10	55 ± 9	51 ± 10***##	<0.001
Disease duration	7 ± 5	8 ± 6	0.017	8 ± 5	9 ± 5*	8 ± 7	0.068
H&Y	2.1 ± 0.6	2.2 ± 0.7	0.466^a^	2.2 ± 0.8	2.4 ± 0.6	2.1 ± 0.6	0.513^a^
UPDRS-III	26 ± 11	27 ± 12	0.711^a^	28 ± 11	30 ± 15	23 ± 10	0.200^a^
Montreal cognitive assessment	25 ± 4	25 ± 5	0.173^a^	24 ± 4	25 ± 5	24 ± 4	0.280^a^
Levodopa equivalent daily dose	560 ± 451	644 ± 438	0.737^a^	610 ± 357	637 ± 278	709 ± 626	0.783^a^
Cognitive impairment, % (*n*)							
-At baseline	36 (140)	40 (39)	0.482	44 (21)	25 (5)	43 (13)	0.397
-Development during study	27 (57)	28 (15)	0.865	24 (6)	21 (3)	40 (6)	0.653
Eotaxin-1	57.0 ± 7.77	57.8 ± 7.37	0.476^a^	57.6 ± 7.72	58.8 ± 5.29	57.4 ± 8.13	0.785^a^
Factor-VII	437 ± 199	476 ± 204	0.103^a^	470 ± 213	475 ± 176	487 ± 212	0.427^a^
Intercellular adhesion molecule 1	511 ± 181	504 ± 183	0.831^a^	511 ± 168	554 ± 199	459 ± 190	0.586^a^
Interleukin-1 receptor antagonist	19.3 ± 8.64	20.32 ± 10.15	0.408^a^	19.6 ± 9.97	21.8 ± 9.26	20.7 ± 11.5	0.695^a^
Interleukin-2	10.7 ± 5.74	11.03 ± 6.52	0.456^a^	10.4 ± 4.88	12.4 ± 8.09	11.2 ± 7.87	0.560^a^
Interleukin-4	8.12 ± 4.03	8.906 ± 4.99	0.309^a^	8.48 ± 5.02	9.34 ± 5.23	9.28 ± 5.02	0.745^a^
Interleukin-6	1.04 ± 0.710	1.011 ± 0.381	0.701^a^	1.03 ± 0.44	1.01 ± 0.37	0.98 ± 0.26	0.959^a^
Interleukin-7	4.18 ± 2.51	4.47 ± 2.84	0.591^a^	4.27 ± 2.99	3.63 ± 1.85	5.45 ± 3.11	0.317^a^
Interleukin-8	40.5 ± 19.4	40.3 ± 12	0.881^a^	39.8 ± 11.5	44.4 ± 12.4	38.4 ± 12.1	0.812^a^
Interleukin-12p40	0.12 ± 0.06	0.12 ± 0.06	0.811^a^	0.11 ± 0.05	0.13 ± 0.08	0.13 ± 0.06	0.559^a^
Interleukin-18	8.26 ± 3.94	9.00 ± 4.49	0.137^a^	9.16 ± 5.03	7.86 ± 3.63	9.48 ± 4.13	0.240^a^
Macrophage inflammatory protein 1 alpha	10.4 ± 4.88	10.0 ± 4.78	0.827^a^	10.3 ± 5.37	10.9 ± 5.20	9.32 ± 3.98	0.967^a^
Macrophage inflammatory protein 1 beta	64.0 ± 19.0	65.4 ± 18.2	0.413^a^	68.2 ± 20.8	61.7 ± 13.5	63.3 ± 16.0	0.442^a^
Matrix metallopeptidase 3	151 ± 65	137 ± 68	0.084^a^	138 ± 62	148 ± 58	130 ± 82	0.310^a^
Matrix metallopeptidase 9	6895 ± 3453	6430 ± 3128	0.234^a^	6706 ± 2998	6568 ± 3438	5839 ± 3190	0.393^a^
Monocyte chemoattractant Protein 1	682 ± 232	712 ± 216	0.204^a^	707 ± 247	764 ± 196	686 ± 174	0.416^a^
Stem cell factor	74.7 ± 23.9	74.8 ± 27.7	0.631^a^	79.5 ± 25.3	71.6 ± 26.1	69.5 ± 31.9	0.474^a^
Tumour necrosis factor-alpha	3.72 ± 1.32 *n* = 128	3.14 ± 0.92 *n* = 46	0.003^a^	3.07 ± 1.15* *n* = 20	3.27 ± 0.85 *n* = 11	3.13 ± 0.61 *n* = 15	0.026^a^
Vascular endothelial growth Factor	35.6 ± 6.14	36.5 ± 5.93	0.192^a^	36.37 ± 6.27	37.1 ± 5.72	36.2 ± 5.67	0.565^a^

Data were presented as mean and standard deviation.

^a^ANCOVA: age and disease duration as a covariate.

^b^*p* value comparing all four groups of GBA_WT_, GBA_risk_, GBA_mild_ and GBA_severe._

*versus GBA_WT_, # versus GBA_risk_ and § versus GBA_mild._

### Correlations between CSF inflammatory markers and clinical data

#### Parkinson´s disease

In males, higher levels of Interleukin-8 (*p* = 0.020), Interleukin-18 (*p* = 0.029), MCP-1 (*p* = 0.019) and MIP-1 beta (*p* = 0.011) were associated with lower MoCA scores whereas higher levels of Interleukin-2 (*p* = 0.039) were associated with higher MoCA scores. Higher levels of Eotaxin-1 were associated with higher UPDRS-III scores (*p* = 0.036). Moreover, higher levels of Interleukin-8 (*p* = 0.027), MIP-1 beta (*p* = 0.005) and SCF (*p* = 0.006) were associated with higher LEDD.

In females, higher levels of ICAM-1 (*p* < 0.001), Interleukin-8 (*p* = 0.002), MMP3 (*p* = 0.035) and VEGF (*p* = 0.032) were associated with lower MoCA scores. Higher levels of ICAM-1 (*p* = 0.005) and VEGF (*p* = 0.004) were associated with higher H&Y staging. Higher levels of Interleukin-8 (*p* = 0.020), MCP-1 (*p* = 0.009) and VEGF (*p* = 0.009) were associated with higher UPDRS-III scores. Higher levels of Interleukin-4 (*p* = 0.009), Interleukin-8 (*p* = 0.044) and VEGF (*p* = 0.023) were associated with higher LEDD.

All correlation coefficients of these significant associations were between 0.11 and 0.36, see Table 2. There were no significant correlations of Factor-VII, MMP9, Interleukin-6, Interleukin-7 and Interleukin-1ra with any of the clinical measures.

**Table 2:** Correlation between CSF inflammatory markers with demographic and clinical characteristics in people with Parkinson´s disease.

	Age	Age at onset	Disease duration	H&Y	UPDRS-III	MoCA	LEDD
Eotaxin-1	n.s.	n.s.	n.s.	n.s.	m: 0.121* *n* = 300	n.s.	n.s.
ICAM-1	t: 0.241*** *n* = 486 m: 0.180*** *n* = 313 f: 0.374*** *n* = 173	t: 0.161*** *n* = 486 m: 0.125* *n* = 313 f: 0.238** *n* = 173	t: 0.137** *n* = 486 f: 0.205** *n* = 173	t: 0.114* *n* = 484 f: 0.211** *n* = 173	n.s.	t: −0.192*** *n* = 462 f: −0.355*** *n* = 168	n.s.
Interleukin-2	t: 0.108* *n* = 359	t: 0.111* *n* = 359	n.s.	n.s.	n.s.	m: 0.138* n = 223	n.s.
Interleukin-4	t: -0.118* *n* = 300	t: −0.175** *n* = 300 m: −0.150* *n* = 191 f: −0.226* *n* = 109	t: 0.128* *n* = 300 f: 0.259** *n* = 109	n.s.	n.s.	n.s.	t: 0.126* *n* = 292 f: 0.252** *n* = 105
Interleukin-8	t: 0.130** *n* = 492 m: 0.194*** *n* = 316	m: 0.134* *n* = 316	t: 0.130** *n* = 492 f: 0.160* *n* = 176	n.s.	f: 0.183* *n* = 162	t: −0.166*** *n* = 467 m: −0.134* *n* = 297 f: −0.234** *n* = 170	t: 0.138* *n* = 479 m: 0.125* *n* = 310 f: 0.155* *n* = 169
Interleukin-12p40	n.s.	n.s.	n.s.	n.s.	t: 0.108* *n* = 377	t: −0.125* *n* = 383	n.s.
Interleukin-18	n.s.	n.s.	n.s.	n.s.	n.s.	m: −0.142* *n* = 234	n.s.
MCP-1	t: 0.177*** *n* = 492 m: 0.185*** *n* = 316 f: 0.194** *n* = 176	t: 0.125** *n* = 492 m: 0.164** *n* = 316	f: 0.217** *n* = 176	n.s.	f: 0.206** *n* = 162	t: −0.133** *n* = 467 m: −0.136* *n* = 297	n.s.
MIP-1 alpha	t: 0.282*** *n* = 199 m: 0.203* *n* = 128 f: 0.408*** *n* = 71	t: 0.194** *n* = 199 f: 0.297* *n* = 71	t: 0.140* *n* = 199	n.s.	n.s.	n.s.	n.s.
MIP-1 beta	t: 0.226*** *n* = 494 m: 0.207*** *n* = 318 f: 0.269*** *n* = 176	t: 0.138** *n* = 494 m: 0.134* *n* = 318	t: 0.153*** *n* = 494 m: 0.135* *n* = 318 f: 0.191* *n* = 176	n.s.	n.s.	t: −0.117** *n* = 469 m: −0.147** *n* = 299	t: 0.129** *n* = 481 m: 0.157** *n* = 312
MMP3	t: 0.253*** *n* = 487 m: 0.234*** *n* = 315 f: 0.328*** *n* = 172	t: 0.183*** *n* = 487 m: 0.179*** *n* = 315 f: 0.207** *n* = 172	t: 0.112* *n* = 487 f: 0.176* *n* = 172	n.s.	n.s.	f: −0.163* *n* = 166	n.s.
SCF	t: 0.266*** *n* = 493 m: 0.244*** *n* = 319 f: 0.303*** *n* = 174	t: 0.200*** *n* = 493 m: 0.176** *n* = 319 f: 0.241*** *n* = 174	t: 0.100* *n* = 493 m: 0.117* *n* = 319	n.s.	n.s.	n.s.	t: 0.114* *n* = 480 m: 0.154** *n* = 313
TNF-alpha	n.s.	n.s.	f: 0.264* *n* = 62	n.s.	n.s.	n.s.	n.s.
VEGF	t: 0.197*** *n* = 495 m: 0.191*** *n* = 320 f: 0.236** *n* = 175	t: 0.100* *n* = 495 m: 0.115* *n* = 320	t: 0.178*** *n* = 495 m: 0.144** *n* = 320 f: 0.256*** *n* = 175	t: 0.103* *n* = 493 f: 0.216** *n* = 175	t: 0.102* *n* = 462 f: 0.204** *n* = 162	f: −0.165* *n* = 169	t: 0.139** *n* = 482 f: 0.175* *n* = 168

Pearson correlation: *p < 0.05; **p < 0.01; ***p ≤ 0.001.

*t* total cohort, *m* males only, *f* females only, *n.s*. not significant.

#### Dementia with Lewy bodies

In males, lower levels of Interleukin-18 were associated with higher H&Y scores (*p* = 0.038). Moreover, lower levels of MIP-1-beta were associated with higher UPDRS-III scores (*p* = 0.041). Higher levels of Interleukin-12p40 were associated with lower LEDD (*p* = 0.028), while higher levels of MCP-1 (*p* = 0.035) and VEGF (*p* = 0.021) were associated with higher LEDD.

In females, higher levels of MMP3 (*p* = 0.040) were associated with lower MoCA scores, while higher levels of Interleukin-7 (*p* = 0.009), Interleukin-18 (*p* = 0.034), and MCP-1 (*p* = 0.014) were associated with higher MoCA scores. Higher levels of MMP3 were associated with higher H&Y staging (*p* = 0.033), while higher levels of VEGF were associated with lower H&Y staging (*p* = 0.018).

All correlation coefficients of these significant associations were between 0.353 and 0.747, see Supplemental Table 1.

### Correlation between CSF inflammatory markers with markers for neurodegeneration in PD and DLB

#### Parkinson’s disease

In males, higher levels of ICAM-1 (*p* = 0.020), Interleukin-8 (*p* = 0.018), MIP-1 beta (*p* = 0.009), MMP3 (*p* < 0.001), SCF (*p* < 0.001) and VEGF (*p* < 0.001) were associated with higher CSF levels of Aβ_1-42_. Higher levels of ICAM-1 (*p* < 0.001), Interleukin-8 (*p* = 0.010), MCP-1 (*p* = 0.002), MIP-1 beta (*p* = 0.009), MMP3 (*p* < 0.001), SCF (*p* < 0.001) and VEGF (*p* < 0.001) were associated with higher CSF levels of t-Tau. Higher levels of ICAM-1 (*p* < 0.001), MCP-1 (*p* = 0.038), MIP-1 beta (*p* < 0.001), MMP3 (*p* < 0.001), SCF (*p* < 0.001) and VEGF (*p* < 0.001) were associated with higher CSF levels of p181-Tau. Higher levels of ICAM-1 (*p* = 0.001), Interleukin-6 (*p* = 0.002), Interleukin-8 (*p* < 0.001), MCP-1 (*p* = 0.003), MIP-1 beta (*p* = 0.019), MMP3 (*p* < 0.001), SCF (*p* = 0.002) and VEGF (*p* = 0.005) were associated with higher levels of NFL. Higher levels of ICAM-1 (*p* < 0.001), MIP-1 beta (*p* < 0.001), MMP3 (*p* < 0.001), SCF (*p* < 0.001) and VEGF (*p* = 0.009) were associated with higher levels of α-synuclein. All correlation coefficients of these significant associations were between 0.134 and 0.612.

In females, higher levels of Interleukin-6 (*p* = 0.040), Interleukin-8 (*p* = 0.015), Interleukin-18 (*p* = 0.024), MCP-1 (*p* = 0.044), SCF (*p* = 0.016) and VEGF (*p* = 0.005) were associated with higher CSF levels of Aβ_1-42_. Higher levels of ICAM-1 (*p* < 0.001), Interleukin-8 (*p* = 0.042), MIP-1 beta (*p* < 0.001), MMP3 (*p* < 0.001), SCF (*p* < 0.001), TNF-alpha (*p* = 0.002) and VEGF (*p* = 0.002) were associated with higher CSF levels of t-Tau. Higher levels of ICAM-1 (*p* < 0.001), MIP-1 beta (*p* < 0.001), MMP3 (*p* < 0.001), SCF (*p* < 0.001), TNF-alpha (*p* = 0.027) and VEGF (*p* = 0.012) were associated with higher CSF levels of p181-Tau. Higher levels of ICAM-1 (*p* = 0.002), Interleukin-8 (*p* = 0.045) and SCF (*p* = 0.002) were associated with higher levels of NFL. Higher levels of ICAM-1 (*p* = 0.002), MIP-1 beta (*p* < 0.001), MMP3 (*p* < 0.001) and SCF (*p* < 0.001) were associated with higher levels of α-synuclein. All correlation coefficients of these significant associations were between 0.154 and 0.539. Table 3. There were no significant correlations of Eotaxin-1, Factor-VII, Interleukin-2, Interleukin-4, Interleukin-7, Interlukin-12p40 and Interleukin-1ra with any of the CSF markers in males or females.

**Table 3:** Correlation between CSF inflammatory markers with CSF PD-biomarkers in people with Parkinson´s disease.

	Aβ_1-42_	t-Tau	p181-Tau	NFL	α-synuclein
ICAM-1	t: 0.101* *n* = 470 m: 0.134* *n* = 301	t: 0.278*** *n* = 470 m: 0.274*** *n* = 301 f: 0.320*** *n* = 169	t: 0.217*** *n* = 463 m: 0.195*** *n* = 298 f: 0.277*** *n* = 165	t: 0.211*** *n* = 445 m: 0.188*** *n* = 287 f: 0.245** *n* = 158	t: 0.211*** *n* = 454 m: 0.222*** *n* = 290 f: 0.236** *n* = 164
Interleukin-4					t: 0.127* *n* = 299
Interleukin-6	f: 0.214* *n* = 92	n.s.	n.s.	m: 0.224** *n* = 193	
Interleukin-8	t: 0.154*** *n* = 475 m: 0.135* *n* = 304 f: 0.186* *n* = 171	t: 0.143** *n* = 475 m: 0.147** *n* = 304 f: 0.156* *n* = 171	t: 0.105* *n* = 468	t: 0.194*** *n* = 451 m: 0.241*** *n* = 291 f: 0.159* *n* = 160	n.s.
Interleukin-18	f: 0.206* *n* = 121	n.s.	n.s.	n.s.	n.s.
MCP-1	t: 0.121** *n* = 475 f: 0.154* *n* = 177	t: 0.141** *n* = 475 m: 0.181** *n* = 304	t: 0.105* *n* = 468 m: 0.120* *n* = 304	t: 0.124** *n* = 451 m: 0.174** *n* = 291	n.s.
MIP-1 alpha	n.s.	n.s.	n.s.	n.s.	t: 0.171* *n* = 182
MIP-1 beta	t: 0.140*** *n* = 477 m: 0.150** *n* = 306	t: 0.279*** *n* = 477 m: 0.234*** *n* = 306 f: 0.373*** *n* = 171	t: 0.261*** *n* = 470 m: 0.213*** *n* = 303 f: 0.362*** *n* = 167	m: 0.137* *n* = 292	t: 0.217*** *n* = 461 m: 0.193*** *n* = 295 f: 0.279*** *n* = 167
MMP3	t: 0.212*** *n* = 471 m: 0.274*** *n* = 303	t: 0.355*** *n* = 471 m: 0.350*** *n* = 303 f: 0.439*** *n* = 168	t: 0.382*** *n* = 464 m: 0.387*** *n* = 300 f: 0.456*** *n* = 164	t: 0.187*** *n* = 446 m: 0.229*** *n* = 289	t: 0.308*** *n* = 455 m: 0.297*** *n* = 292 f: 0.424*** *n* = 163
MMP9	n.s.	m: −0.124* *n* = 271	n.s.	n.s.	n.s.
SCF	t: 0.204*** *n* = 478 m: 0.216*** *n* = 308 f: 0.184* *n* = 170	t: 0.464*** *n* = 478 m: 0.439*** *n* = 308 f: 0.502*** *n* = 170	t: 0.469*** *n* = 471 m: 0.421*** *n* = 305 f: 0.539*** *n* = 166	t: 0.198*** *n* = 453 m: 0.178** *n* = 294 f: 0.239** *n* = 159	t: 0.448*** *n* = 461 m: 0.440*** *n* = 296 f: 0.475*** *n* = 165
TNF-alpha	n.s.	f: 0.402** *n* = 59	f: 0.293* *n* = 57	n.s.	n.s.
VEGF	t: 0.222*** *n* = 478 m: 0.226*** *n* = 308 f: 0.215** *n* = 170	t: 0.198*** *n* = 478 m: 0.206*** *n* = 308 f: 0.237** *n* = 170	t: 0.184*** *n* = 471 m: 0.207*** *n* = 305 f: 0.195* *n* = 166	t: 0.139** *n* = 454 m: 0.162** *n* = 294	t: 0.121** *n* = 463 m: 0.151** *n* = 297

Pearson correlation: *p < 0.05; **p < 0.01; ***p ≤ 0.001.

*t* total cohort, *m* males only, *f* females only, *n.s.* not significant.

#### Dementia with Lewy bodies

In males, higher levels of Eotaxin-1 (*p* = 0.032), Interleukin-8 (*p* = 0.031), and SCF (*p* = 0.010) were associated with higher CSF levels of t-Tau. Higher levels of Interleukin-8 (*p* = 0.006), Interleukin-18 (*p* = 0.031), MMP3 (*p* = 0.039) and SCF (*p* < 0.001) were associated with higher CSF levels of p181-Tau. Higher levels of ICAM-1 (*p* < 0.001), MMP3 (*p* = 0.030) and VEGF (*p* < 0.001) were associated with higher levels of NFL. Higher levels of MMP9 (*p* = 0.042) and SCF (*p* < 0.001) were associated with higher levels of α-synuclein. All correlation coefficients of these significant associations were between 0.320 and 0.649.

In females, higher levels of Interleukin-4 were associated with higher CSF levels of Aβ_1-42_ (*p* = 0.037). Higher levels of Factor-VII (*p* = 0.032), Interleukin-8 (*p* = 0.009), MMP3 (*p* = 0.037) and SCF (*p* < 0.001) were associated with higher CSF levels of t-Tau. Higher levels of Factor-VII (*p* = 0.022), Interleukin-8 (*p* < 0.001) and SCF (*p* < 0.001) were associated with higher CSF levels of p181-Tau. Higher levels of Interleukin-4 (*p* = 0.004) and Interleukin-6 (*p* = 0.020) were associated with higher levels of NFL. Higher levels of Factor-VII (*p* = 0.041), Interleukin-8 (*p* = 0.027), MMP3 (*p* = 0.007) and SCF (*p* < 0.001) were associated with higher levels of α-synuclein. All correlation coefficients of these significant associations were between 0.458 and 0.801, Table 4. There were no significant correlations of Interleukin-2, Interleukin-7, Interleukin-12p40, Interleukin-1ra, MIP-1 alpha, MIP-1 beta and TNF-alpha with any of the CSF markers in males or females.

**Table 4:** Correlation between CSF inflammatory markers with CSF PD-biomarkers in people with dementia with Lewy bodies.

	Aβ_1-42_	t-Tau	p181-Tau	NFL	α-synuclein
Eotaxin-1	n.s.	m: 0.321* *n* = 45	n.s.	n.s.	n.s.
Factor-VII	n.s.	f: 0.572* *n* = 14	f: 0.604* *n* = 14	n.s.	f: 0.552* *n* = 14
ICAM-1	n.s.	n.s.	n.s.	t: 0.457*** *n* = 61 m: 0.649*** *n* = 41	n.s.
Interleukin-4	f: 0.495* *n* = 18	n.s.	n.s.	f: 0.666** *n* = 17	n.s.
Interleukin-6	n.s.	n.s.	n.s.	t: 0.341* *n* = 45 f: 0.656* *n* = 12	n.s.
Interleukin-8	n.s.	t: 0.407*** *n* = 66 m: 0.322* *n* = 45 f: 0.558** *n* = 21	t: 0.513*** *n* = 62 m: 0.420** *n* = 42 f: 0.719*** *n* = 20	n.s.	t: 0.328** *n* = 62 f: 0.493* *n* = 20
Interleukin-18	n.s.	n.s.	m: 0.359* *n* = 36	n.s.	n.s.
MCP-1	n.s.	t: 0.273* *n* = 66	n.s.	n.s.	n.s.
MMP3	n.s.	f: 0.458* *n* = 21	t: 0.342** *n* = 62 m: 0.320* *n* = 42	t: 0.298* *n* = 61 m: 0.339* *n* = 41	t: 0.361** *n* = 62 f: 0.586** *n* = 20
MMP9	n.s.	n.s.	n.s.	n.s.	t: 0.302* *n* = 55 m: 0.328* *n* = 39
SCF	n.s.	t: 0.550*** *n* = 66 m: 0.380** *n* = 45 f: 0.801*** *n* = 21	t: 0.604*** *n* = 62 m: 0.545*** *n* = 42 f: 0.772** *n* = 20	n.s.	t: 0.645*** *n* = 62 m: 0.573*** *n* = 42 f: 0.759*** *n* = 20
VEGF	n.s.	n.s.	n.s.	t: 0.300* *n* = 61 m: 0.625*** *n* = 41	n.s.

Pearson correlation: *p < 0.05; **p < 0.01; ***p ≤ 0.001.

*t* total cohort, *m* males only, *f* females only, *n.s*. not significant.

## Longitudinal

### Association between CSF inflammatory markers with the clinical endpoint cognitive impairment in PD

Of the 400 PD_GBA_WT_ patients, 140 had cognitive impairment at baseline. Of those without cognitive impairment at baseline, 213 (82%) were followed longitudinally. Of these, 57 patients (27%) developed cognitive impairment during the study, while 156 remained without cognitive impairment. Patients developing cognitive impairment during the study reached this endpoint at a mean study time of 3.8 years with a mean disease duration of 11.0 years. Patients without the development of cognitive impairment were followed-up for a mean study time of 4.4 years until a mean disease duration of 10.9 years (Study time *p* = 0.119; Disease duration *p* = 0.936).

Of the 98 PD_GBA_ patients, 39 had cognitive impairment at baseline. Of those without cognitive impairment at baseline, 54 (92%) were followed longitudinally. Of these, 15 patients (PD_GBA_all_ 28%; PD_GBA_risk_ 24%, PD_GBA_mild_ 21% and PD_GBA_severe_ 40%) developed cognitive impairment during the study, while 39 patients remained without cognitive impairment. Patients developing cognitive impairment during the study reached this endpoint at a mean study time of 3.7 years with a mean disease duration of 10.2 years. Patients without the development of cognitive impairment were followed-up for a mean study time of 4.2 years until a mean disease duration of 11.2 years (Study time *p* = 0.532; Disease duration *p* = 0.547).

Patients with cognitive impairment at baseline had higher levels of Interleukin-12p40 compared to patients developing cognitive impairment during the study and compared to patients without the development of cognitive impairment. Patients who developed cognitive impairment during the study had higher levels of TNF-alpha at baseline (4.10 pg/mL) compared to patients without the development of cognitive impairment (3.42 pg/mL) and compared to patients who presented with cognitive impairment already at baseline (3.53 pg/mL), *p* = 0.037, Table 5.

**Table 5:** Baseline demographic, clinical and inflammatory data from all people with Parkinson´s disease stratified by cognitive status.

	PD no CI during study	PD new CI during study	PD CI at baseline	*p* value PD groups	DLB for descriptive purposes
	*n* = 232	*n* = 72	*n* = 178		*n* = 67
Male, % (*n*)	65 (150)	57 (41)	66 (118)	0.367	69 (46)
Age, years	61 ± 10	64 ± 9^#^	71 ± 8^###§§§^	<0.001	72 ± 6
Age at onset, years	55 ± 11	57 ± 9	62 ± 10^###§§^	<0.001	69 ± 7
Disease duration, years	6 ± 5	7 ± 4	8 ± 5^###^	0.001	4 ± 9
H&Y	2.0 ± 0.5	2.0 ± 0.5	2.3 ± 0.7	0.161^a^	2.4 ± 0.6
UPDRS-III	24 ± 10	27 ± 13^#^	29 ± 12	0.070^a^	25 ± 8
MoCA	27 ± 2	27 ± 2	20 ± 4^###§§§^	<0.001^a^	14 ± 4
LEDD	548 ± 505	666 ± 432^#^	572 ± 374	0.044^a^	375 ± 210
Amyloidβ_1-42_ [pg/ml]	718 ± 245	732 ± 221	689 ± 297	0.481^a^	491 ± 220
total-Tau [pg/ml]	226 ± 100	221 ± 96	272 ± 152	0.371^a^	304 ± 192
phospho181-Tau [pg/ml]	40 ± 16	41 ± 14	45 ± 19	0.607^a^	45 ± 25
NFL [pg/ml]	914 ± 989	835 ± 570	1202 ± 946	0.403^a^	1669 ± 1698
α-synuclein [pg/ml]	591 ± 286	616 ± 311	636 ± 310	0.118^a^	501 ± 292
Eotaxin-1	57.7 ± 7.5	57.4 ± 7.8	56.5 ± 8.0	0.651^a^	55.4 ± 5.8
Factor-VII	450 ± 220	460 ± 200	430 ± 190	0.755^a^	421 ± 186
ICAM-1	430 ± 170	510 ± 210	540 ± 190	0.726^a^	511 ± 224
Interleukin-1ra	19.7 ± 8.8	17.6 ± 7.9	20.1 ± 9.7	0.224^a^	19.8 ± 9.6
Interleukin-2	10.9 ± 6.2	11.4 ± 5.7	10.5 ± 5.5	0.189^a^	10.7 ± 6.2
Interleukin-4	8.73 ± 4.31	7.71 ± 3.18	8.05 ± 3.66	0.372^a^	9.36 ± 4.19
Interleukin-6	1.00 ± 0.41	1.05 ± 0.36	0.98 ± 0.37	0.684^a^	1.12 ± 0.48
Interleukin-7	4.08 ± 2.33	4.33 ± 2.64	4.37 ± 2.81	0.466^a^	4.19 ± 2.76
Interleukin-8	39.5 ± 12.4	37.5 ± 10.5	41.9 ± 14.6	0.247^a^	47.0 ± 16.9
Interleukin-12p40	0.12 ± 0.05	0.11 ± 0.04	0.13 ± 0.07##§§	0.004^a^	0.11 ± 0.04
Interleukin-18	7.87 ± 3.62	9.00 ± 4.99	8.93 ± 4.13	0.130^a^	9.99 ± 4.95
MCP-1	668 ± 221	668 ± 208	719 ± 232	0.716^a^	836 ± 242
MIP-1 alpha	10.2 ± 4.6	9.5 ± 4.8	11.0 ± 5.2	0.622^a^	10.3 ± 4.1
MIP-1 beta	62.6 ± 17.2	62.5 ± 17.6	67.3 ± 20.8	0.814^a^	66.4 ± 13.9
MMP3	140 ± 60	140 ± 60	160 ± 70	0.758^a^	154 ± 76
MMP9	6510 ± 3440	6960 ± 3030	7210 ± 3440	0.064^a^	6047 ± 3355
SCF	73.3 ± 24.4	71.7 ± 24.4	78.8 ± 25.0	0.560^a^	75.2 ± 27.3
TNF-alpha	3.42 ± 1.06	4.10 ± 1.91#	3.53 ± 1.06§	0.037^a^	3.91 ± 1.32
VEGF	35.3 ± 5.9	35.6 ± 5.5	36.7 ± 6.4	0.957^a^	36.8 ± 6.7

Data were presented as mean and standard deviation.

*CI* cognitive impairment.

^a^ANCOVA with age and disease duration as co-variables.

# compared to PD no CI, § compared to PD new CI.

In PD_GBA_ patients, there was no difference in any inflammatory marker between individuals with cognitive impairment at baseline, individuals developing cognitive impairment during the study and individuals without cognitive impairment, Table 6.

**Table 6:** Baseline demographic, clinical and inflammatory data from people with Parkinson´s disease with GBA mutation stratified by cognitive status.

	PD_GBA_ no CI during study	PD_GBA_ new CI during study	PD_GBA_ CI at baseline	*p* value PD groups
	*n* = 44	*n* = 15	*n* = 39	
GBA severity, % (*n*)	Risk 48 (21) Mild 27 (12) Severe 25 (11)	Risk 40 (6) Mild 20 (3) Severe 40 (6)	Risk 40 (21) Mild 13 (5) Severe 33 (13)	
Male, % (*n*)	71 (31)	53 (8)	69 (27)	0.450
Age, years	58 ± 10	66 ± 6	63 ± 9	<0.001
Age at onset, years	51 ± 11	60 ± 8	57 ± 8	0.003
Disease duration, years	7 ± 5	6 ± 3	10 ± 6	0.014
H&Y	2.0 ± 0.5	2.0 ± 0.5	2.4 ± 0.8	0.659^a^
UPDRS-III	25 ± 10	28 ± 14	29 ± 13	0.824^a^
MoCA	28 ± 2	27 ± 1	20 ± 4	<0.001^a^
LEDD	598 ± 498	826 ± 456	526 ± 335	0.021^a^
Amyloidβ_1-42_ [pg/ml]	709 ± 258	694 ± 221	710 ± 254	0.851^a^
total-Tau [pg/ml]	230 ± 89	219 ± 79	272 ± 183	0.381^a^
phospho181-Tau [pg/ml]	40 ± 15	39 ± 14	40 ± 18	0.274^a^
NFL [pg/ml]	815 ± 675	775 ± 321	1059 ± 593	0.616^a^
α-synuclein [pg/ml]	538 ± 236	609 ± 347	529 ± 236	0.183^a^
Eotaxin-1	58.0 ± 6.8	58.0 ± 5.9	57.4 ± 8.6	0.981^a^
Factor-VII	499 ± 250	462 ± 150	456 ± 163	0.771^a^
ICAM-1	482 ± 162	465 ± 156	541 ± 208	0.752^a^
Interleukin-1ra	20.7 ± 10.6	20.2 ± 10.4	20.0 ± 9.9	0.977^a^
Interleukin-2	10.7 ± 6.4	11.4 ± 7.5	11.3 ± 6.5	0.985^a^
Interleukin-4	9.43 ± 5.50	7.08 ± 2.88	9.08 ± 5.09	0.613^a^
Interleukin-6	0.99 ± 0.34	0.98 ± 0.28	1.04 ± 0.46	0.849^a^
Interleukin-7	3.85 ± 2.04	4.25 ± 2.78 *n* = 7	5.13 ± 3.43	0.087^a^
Interleukin-8	38.7 ± 12.4	36.2 ± 6.9	43.7 ± 12.3	0.368^a^
Interleukin-12p40	0.11 ± 0.05	0.09 ± 0.02	0.14 ± 0.07	0.127^a^
Interleukin-18	8.23 ± 4.9	8.93 ± 4.43	9.78 ± 4.87	0.485^a^
MCP-1	668 ± 230	741 ± 179	750 ± 210	0.887^a^
MIP-1 alpha	9.5 ± 3.8	9.3 ± 5.0	10.7 ± 5.6	0.565^a^
MIP-1 beta	62.6 ± 17.5	66.9 ± 16.3	68.0 ± 19.5	0.945^a^
MMP3	128 ± 53	132 ± 51	150 ± 86	0.963^a^
MMP9	6249 ± 2960	6052 ± 2534	6771 ± 3533	0.361^a^
SCF	69.6 ± 27.5	76.9 ± 23.7	80.0 ± 29.0	0.909^a^
TNF-alpha	2.97 ± 0.74	2.97 ± 0.44 *n* = 8	3.40 ± 1.19	0.471^a^
VEGF	35.0 ± 5.7	36.4 ± 6.0	38.1 ± 5.8	0.525^a^

Data were presented as mean and standard deviation.

*CI* cognitive impairment.

^a^ANCOVA with age and disease duration as co-variable.

#### Kaplan–Meier and Cox-regression analyses in people with PD and GBA wildtype status

In PD_GBA_WT_ patients, Kaplan–Meier analyses revealed interleukin-8 to have a significant impact on the duration until the development of cognitive impairment. Fifty percent of patients in the highest inflammatory quartile of Interleukin-8 levels reached this endpoint after 19.2 years of median disease duration compared to 13.1 years of median disease duration in patients in the lowest inflammatory quartile (*p* = 0.019).

**Fig. 1:**
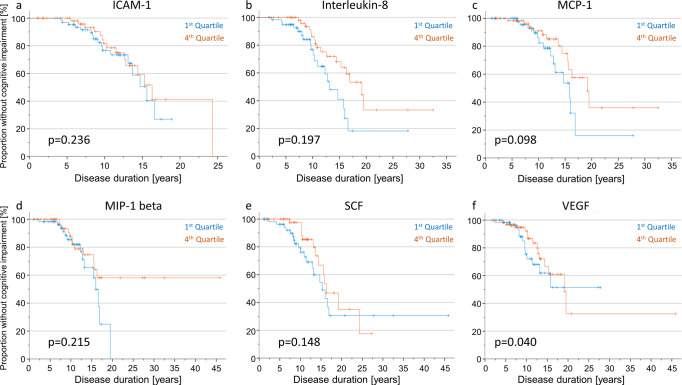
Kaplan–Meier curves and Cox regression analyses depicting the duration until the development of cognitive impairment in PD_GBA_WT_ people stratified by inflammatory profiles. **a**–**f** Kaplan–Meier analyses of the most relevant inflammatory markers (ICAM-1, interleukin-8, MCP-1, MIP-1 beta, SCF and VEGF) revealed interleukin-8 to have a significant impact on the duration until the development of cognitive impairment. Fifty percent of patients in the highest inflammatory quartile of interleukin-8 levels reached this endpoint after 19 years of disease duration compared to 13 years in patients in the lowest inflammatory quartile (*p* = 0.019). However, adjusting the model for age, sex, and disease duration as additional interacting co-variates, only VEGF showed a significant effect on the duration until the development of cognitive impairment in Cox regression analyses. Fifty percent of patients with high CSF levels of VEGF at baseline (fourth quartile) reached this clinical endpoint after 19 years compared to 16 years in patients with low levels (first quartile).

Cox-regression analyses revealed a relevant impact of age and/or disease duration as confounding interaction with the respective inflammatory marker on the duration until the development of cognitive impairment (ICAM-1: age *p* = 0.024, dd *p* = 0.010; interleukin-8: age *p* = 0.024, dd *p* < 0.001; MCP-1: dd *p* < 0.001; MIP-1 beta: dd *p* < 0.001; SCF: dd *p* < 0.001; VEGF: dd *p* < 0.001). We, therefore, modelled the inflammation groups (patients within the highest versus lowest quartile of the respective inflammatory marker) along with age, sex, and disease duration as interacting co-variate and evaluated their impact on the longitudinal development of cognitive impairment. Hereby, Cox regression revealed a significant difference in disease duration until cognitive impairment for levels of VEGF. Fifty percent of patients in the highest inflammatory quartile of VEGF levels reached this endpoint after 19 years, whereas 50% of patients in the lowest inflammatory quartile reached this endpoint after 16 years (*p* = 0.040). A similar trend was seen for MCP-1 but did not reach significance (*p* = 0.098), Fig. 1. Importantly, patients in the highest VEGF quartile presented with a mean age at onset of 57 years, mean age at baseline of 65 years and mean disease duration of 8 years whereas patients in the lowest VEGF quartile had a mean age at onset of 55 years, mean age at baseline of 61 years and a mean disease duration of 6 years (age at onset *p* = 0.133, age *p* = 0.009, disease duration *p* = 0.076).

#### Kaplan–Meier and Cox-regression analyses in people with PD and GBA mutation

In PD_GBA_ patients, Kaplan–Meier analyses revealed no significant effects of inflammatory profiles on the duration until the development of cognitive impairment.

**Fig. 2:**
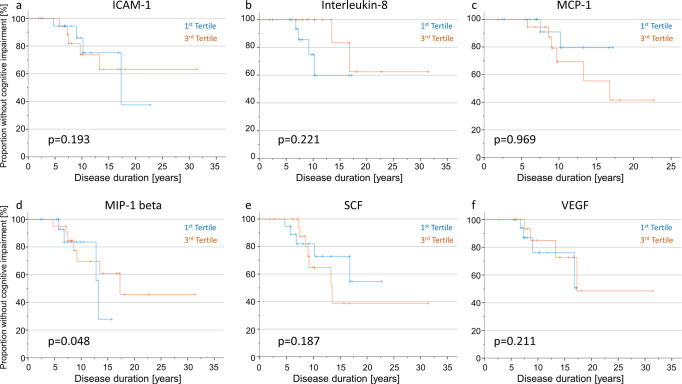
Kaplan–Meier curves and Cox regression analyses depicting the duration until the development of cognitive impairment in PD_GBA_ people stratified by inflammatory profiles. **a**–**f** Kaplan–Meier analyses of the most relevant inflammatory markers (ICAM-1, interleukin-8, MCP-1, MIP-1 beta, SCF and VEGF) revealed no significant impact on the duration until the development of cognitive impairment. However, adjusting the model for age, sex and disease duration as additional interacting co-variates MIP-1-beta showed a significant effect on the duration until the development of cognitive impairment in Cox regression analyses. Fifty percent of patients with high CSF levels of MIP-1-beta at baseline (third tertile) reached this clinical endpoint after 17 years compared to 13 years in patients with low levels (first tertile).

Similar to PD_GBA_WT_, Cox-regression analyses in PD_GBA_ patients revealed an impact of age and/or disease duration as confounding interaction with the respective inflammatory marker on the duration until the development of cognitive impairment (ICAM-1: age *p* = 0.035, dd *p* = 0.053; interleukin-8: dd *p* = 0.076; MCP-1: dd *p* = 0.048, MIP-1 beta: dd *p* = 0.062; SCF: dd *p* = 0.094; VEGF: age *p* = 0.074, dd *p* = 0.040). We, therefore, modelled the inflammation groups (patients within the highest versus lowest tertile of the respective inflammatory marker) along with age, sex, and disease duration as interacting co-variate and evaluated their impact on the longitudinal development of cognitive impairment. Hereby, Cox regression revealed a significant difference in disease duration until cognitive impairment for levels of MIP-1 beta. Fifty percent of patients in the highest tertile of MIP-1 beta levels reached this endpoint after 17.3 years compared to 13.3 years in patients in the lowest tertile (*p* = 0.048), Fig. 2. Importantly, patients in the highest MIP-1 beta tertile presented with a mean age at onset of 56 years, mean age at baseline of 64 years and mean disease duration of 8 years whereas patients in the lowest of MIP-1 beta tertile had a mean age at onset of 54 years, mean age at baseline of 59 years and a mean disease duration of 5 years (age at onset *p* = 0.710, age *p* = 0.112, disease duration *p* = 0.040).

## Discussion

By using a multiplex assay and assessing 30 inflammatory markers in CSF in 498 people with PD and 67 people with DLB, we show:

### Cross-sectionally

(I) Higher CSF levels of ICAM-1, interleukin-8, MCP-1 and MIP-1 beta were associated with lower MoCA scores in the total PD cohort and also after stratification by sex.
(II) Higher CSF levels of ICAM-1, interleukin-8, MMP3, MIP-1 beta, SCF and VEGF were associated with higher CSF levels of neurodegenerative/PD-specific biomarkers, namely Aβ_1-42_, t-Tau, p181-Tau, NFL and α-synuclein in the total PD cohort and also after stratification by sex. Similarly, higher CSF levels of ICAM-1, interleukin-8, MMP3, SCF and VEGF were associated with higher CSF levels of neurodegenerative/PD-specific biomarkers in the DLB cohort.
(III) PD_GBA_ patients show similar levels of inflammatory CSF markers when compared to PD_GBA_WT_ patients, even when stratified by mutation severity.

### Longitudinally

(I) PD patients who developed cognitive impairment during the study had higher CSF levels of TNF-alpha at baseline compared to patients without the development of cognitive impairment and compared to patients who presented with cognitive impairment already at baseline.
(II) Higher CSF levels of VEGF were associated with a longer duration until the development of cognitive impairment in PD_GBA_WT_ patients.
(III) Higher CSF levels of MIP-1 beta were associated with a longer duration until the development of cognitive impairment in PD_GBA_ patients.

Using a different multiplexed immunoassay by Myriad RBM, Austin, TX, USA (http://rbm.myriad.com) with an assessment of 41 inflammatory markers in CSF/serum pairs in 453 sporadic PD patients, we could previously show that the most important inflammatory mediators associated with motor and cognitive function and with neurodegenerative/PD-specific biomarkers were FABP, ICAM-1, IL-8, MCP-1, MIP-1-beta and SCF. At that point, results were more robust for CSF than for serum^21^. Except for FABP, which was not part of the current assay and VEGF, which was not included in the first assay, all other inflammatory markers from the current assay were also included in the Myriad assay. Therefore, the finding of relevant associations of higher CSF levels of ICAM-1, interleukin-8, MCP-1, MIP-1-beta and SCF with cognitive dysfunction and with higher CSF levels of neurodegenerative/PD-specific biomarkers seem consistent in sporadic PD and also relevant when including PD_GBA_ patients and patients with DLB. Other cross-sectional studies in sporadic PD patients reported higher CSF levels of CRP, interleukin-6 and interleukin-8^27,28^ to be associated with worse motor function^28,29^. One longitudinal study found that higher CSF levels of MCP-1 and MMP10 were associated with more severe motor impairment^30^. Similarly, several cross-sectional studies reported higher CSF levels of CRP, interleukin-6, interleukin-8, MCP-1, SCF and YKL-40 to be related to worse cognitive function^28,29,31,32^. Higher CSF levels of CRP and MCP-1 were also associated with depression and fatigue^28,31^. While the type of assays (single vs. multiplex, platforms), assessed cytokines and cohorts’ characteristics (samples size, disease duration) are quite variable, inflammatory markers of the monocyte-macrophage signalling and chemotaxis pathway (ICAM-1, interleukin-8, MCP-1, MIP-1 beta and SCF) seem to play a relevant role in PD-associated inflammation. ICAM-1 has been demonstrated in sustaining neuroinflammation via activated microglia in PD brains, MPTP-treated monkeys and rats^33,34^. Interleukin-8 is produced by macrophages and promotes chemotaxis, causing granulocytes to migrate toward sites of infectious/injured tissue where, as a second function of Interleukin-8, phagocytosis is induced. Secretion of interleukin-8 is increased by oxidative stress, which promotes inflammation and thereby further enhances oxidative stress in a vicious circle. MCP-1 has a chemotactic function on monocytes, while MIP-1 is produced by macrophages and promotes chemotaxis and synthesis of other pro-inflammatory cytokines such as Interleukin-1, Interleukin-6 and TNF-alpha^35^. In vivo and in vitro experiments show an up-regulation of SCF in neurons of injured brain tissue paralleled by neural stem/progenitor cell migration highlighting that SCF is involved in self-renewal and cell survival^36^. A central role in maintaining chronic inflammation upon α-synuclein aggregation and cell death is the bi-directional loop between activated microglia and activated inflammasome^37^. Both further induce the secretion of inflammatory cytokines such as interleukin-1 beta, interleukin-6 and MCP-1. Studies in mouse models of Alzheimer’s disease, brain injury, myocardial infarction and inflammatory bowel disease could demonstrate a reduction in expression levels of interleukin-1 beta, interleukin-6, MCP-1 and NLRP3 upon treatment with pterostilbene and prebiotics^38–41^. These findings offer the chance to accumulate knowledge on mechanistic aspects and treatment options of inflammation across different (neurodegenerative) diseases.

While one small study reported increased plasma levels of IFN-γ, interleukin-1 beta, interleukin-2 and TNF-alpha in eight PD_GBA_ patients^24^, two larger studies did not find any differences in blood and CSF levels between PD patients with versus without heterozygous *GBA* mutations which is in line with our findings^25,26^.

Our finding of a longer duration until the development of cognitive impairment in patients of the highest quartile of VEGF levels is in line with recent studies. VEGF and, more specifically, the members of the VEGF family (VEGF A-D) have several functions. The first recognised was angiogenesis. Additionally, VEGF connects to VEGF receptor-coreceptors of the neuropilin-family and plays a relevant role in several neurodegenerative diseases. Stimulation of the VEGF-receptor pathway leads to enhanced vascular permeability, angiogenesis, neuronal outgrowth and neuroprotection. Specifically, VEGF-receptor 3, which is primarily known for its lymphangiogenesis-promoting effects, regulates neuronal development and adult neuronal function in the central nervous system angiogenesis and axonal guidance. Following this line of reasoning, the application of VEGF showed neuroprotective effects in an in vitro model of PD^42,43^. Moreover, VEGF plays a key role in promoting hippocampal synaptic plasticity and memory consolidation. Studies in mice could show that VEGF overexpression improves cognitive function and memory performance^44,45^ and sustained activity in the hippocampus triggers a rapid release of VEGF, suggesting that activity-dependent secretion of VEGF is involved in synaptic plasticity^46^. In humans, higher CSF levels of VEGF have been associated with increased hippocampal volume and improved cognitive performance over time^47^. Further, if typical Alzheimer’s disease biomarkers signatures in CSF are present, elevated VEGF levels are associated with less cognitive decline^47^ highlighting a potential neuroprotective role for VEGF.

We conclude that CSF levels of inflammatory markers are associated with clinical rating scales of motor and cognitive function as well as with levels of neurodegenerative CSF markers but that the majority of these inflammatory CSF markers is limited in robustly predicting longitudinal trajectories of developing cognitive impairment, at least in our two cohorts of PD patients with and without *GBA* mutation. Further longitudinal analyses in de-novo PD patients with a long follow-up time until clinical endpoints (e.g. cognitive impairment, falls and malignant subtype) are reached in at least 50% of the cohort are needed in order to support our findings.

Importantly, we detected a relevant interaction of sex, age and disease duration with the CSF levels of inflammatory markers in the cross-sectional also in longitudinal analyses. Therefore, we recommend to define age and sex-specific cut-off values for inflammatory markers. This will be highly necessary before planning and interpreting clinical trials.

The strength of the present study is the large monocentric collection of high-quality CSF samples according to standardised procedures, which minimises variance in sample collection and processing, as often seen in multicenter studies.

Limitations of our study are as follows: (I) The single measurement of inflammatory markers limits the evaluation of variation in repeatedly assessed intra-individual measurements. (II) The mean storage time until measurement of inflammatory markers was 6 years which might impact the detectability of markers that are present at low concentrations. (III) Although spanning a wide spectrum between 1–30 years, the heterogeneous disease duration at the study baseline limits homogenous longitudinal data analyses in all patients from diagnosis on. (IV) As this study is exploratory, we did not correct for multiple testing, and therefore, some associations might be less robust. However, results from this study hopefully will guide further analyses. (V) At the time when we designed the analysis plan, we wanted to explore which of the inflammatory markers are most robustly associated with clinical and neurodegenerative CSF markers (correlation analysis), and thereby we wanted to narrow down the number of cytokines for the longitudinal Kaplan–Meier Analysis. We acknowledge that a factor analysis or principal component analysis are interesting alternatives to narrow down/group the number of cytokines and identify underlying inflammatory profiles.

## Methods

### Clinical investigations

All participants were examined by a neurologist specialised in movement disorders. Diagnosis of PD was defined according to UK Brain Bank Society Criteria^48^. Diagnosis of DLB was made according to the DLB consortium revised consensus criteria^49^. Patients were assessed in the dopaminergic ON state. We assessed the severity of motor symptoms using part III of the Unified Parkinson’s disease Rating Scale (UPDRS-III), from 2006 to 2008, the old version and from 2009 on the MDS-UPDRS^50^. The disease stage was categorised by the modified Hoehn and Yahr Scale (H&Y)^51^. Cognitive function was tested using the Montreal Cognitive Assessment (MoCA)^52^ and/or the Mini-Mental Status Examination (MMSE)^53^. Since the MoCA was available only from 2009 on, all previously obtained MMSE scores were converted into MoCA equivalent scores according to a published algorithm^54^. The presence of cognitive impairment was defined as a MoCA score <26.

Genetic screening for *GBA* variants was done by Sanger sequencing of all exons in PD and DLB patients. In the PD patients group, 98 patients carried a *GBA* mutation, and in the DLB patient group, 19 patients carried a *GBA* mutation.

### Collection of CSF samples

Spinal taps for CSF collection were performed between 9.00 AM and 1.00 PM. Samples were taken from the bedside and centrifuged within 60 min and frozen at −80 °C within 90 min after collection. Until 2013, we used polypropylene tubes from Sarstedt (Article Nr. 72.730.406) and from 2013 on, we used low protein-binding polypropylene cryovial 2D barcode cryovials FluidX (Article Nr. 68-0703-01) for storage. Samples with abnormal routine CSF diagnostics (erythrocytes >1/µl, white blood cell count >5 cells/µl, immunoglobulin subtype G index >0.7) were excluded.

### CSF measurement of inflammatory markers

Levels of 30 inflammation-associated markers were measured in CSF using the multiplexed immunoassay provided by Rules-Based Medicine—a Q2 Solutions Company, Austin, TX, USA (https://rbm.q2labsolutions.com/). The mean storage time until measurement was 6 years. For measurements, samples were thawed at room temperature, vortexed, spun at 18,000×*g* for 1 min and pipetted into a master microtiter plate. After dilution with assay diluents in a manner of 1:2, an aliquot of 10 µl diluted sample was introduced into one of the capture microsphere multiplexes, followed by incubation at room temperature for 1 h. Reporter antibodies were added, followed by incubation for an additional hour at room temperature. Streptavidin-phycoerythrin solution was added for development and incubated for 1 h at room temperature. For control purposes, calibrators and controls were included on each microtiter plate. Standard curve, control, and sample QC were performed to ensure proper assay performance. Samples were tested in singles.

#### Lower limit of quantitation (LLOQ)

NMI’s requirement for accuracy is the concentration of an analyte at which the coefficient of variation of replicate standard samples is 30%. LLOQ are as follows: BDNF: 27,7 pg/mL; Eotaxin-1: 37,1 pg/mL; Factor-VII: 788 pg/mL; GM-CSF: 23,9 pg/mL; ICAM-1: 359 pg/mL; IFN-gamma: 1,76 pg/mL, Interleukin-1 alpha: 1,09 pg/mL; Interleukin-1 beta: 3,76 pg/mL; Interleukin-1ra: 31,5 pg/mL; Interleukin-2: 27,2 pg/mL; Interleukin-3: 1,74 pg/mL; Interleukin-4: 14 pg/mL; Interleukin-5: 6,53 pg/mL; Interleukin-6: 1,87 pg/mL; Interleukin-7: 8 pg/mL; Interleukin-8: 2,83 pg/mL; Interleukin-10: 5,19 pg/mL; Interleukin-12p40: 0,174 pg/mL; Interleukin-12p70: 22,8 pg/mL; Interleukin-17: 1,53 pg/mL; Interleukin-18: 16,8 pg/mL; MCP-1: 14,7 pg/mL; MIP-1 alpha: 15,4 pg/mL; MIP-1 beta: 21,1 pg/mL; MMP3: 28 pg/mL; MMP9: 11500 pg/mL; SCF: 43,6 pg/mL; TNF-alpha: 6,52 pg/mL; TNF-beta: 10,2 pg/mL; VEGF: 10,7 pg/mL.

#### Number of total reads per inflammation marker

BDNF 0/565; Eotaxin-1 563/565; Factor-VII 428/565; GM-CSF 1/565; ICAM-1 556/565; IFN-gamma 64/565; Interleukin-1 alpha 31/565; Interleukin-1 beta 7/565; Interleukin-1ra 419/565; Interleukin-2 409/565; Interleukin-3 54/565; Interleukin-4 352/565; Interleukin-5 2/565; Interleukin-6 366/565; Interleukin-7 308/565; Interleukin-8 565/565; Interleukin-10 14/565; Interleukin-12p40 460/565; Interleukin-12p70 7/565; Interleukin-17 34/565; Interleukin-18 420/565; MIP-1 alpha 240/565; MIP-1 beta 565/565; MMP3 563/565; MMP9 492/565; MCP-1 565/565; SCF 561/565; TNF-alpha 204/565; TNF-beta 9/565 and VEGF 563/565.

Of these 30 analyzed inflammatory markers, some were measurable only in a small number of cases (below one-third; <33%) of the total cohort) and were therefore excluded from the analyses due to lack of meaningful outcome: BDNF, GM-CSF, interferon gamma, interleukin-1 alpha, interleukin-1 beta, interleukin-3, interleukin-5, interleukin-10, interleukin-12p70, interleukin-17 and TNF-beta.

### CSF measurement of Aβ_1-42_, t-Tau, p181-Tau, NFL and total α-synuclein

CSF levels of Aβ_1-42_, t-Tau and p181-Tau were measured using ELISA kits from INNOTEST, Fujirebio GmbH, Germany. CSF levels of NFL were measured using the UmanDiagnostics NF-light®assay. Intra-assay coefficients of variation for each CSF parameter were below 15%. CSF levels of total α-synuclein were assessed using an ELISA kit for human α-synuclein (Roboscreen GmbH, Germany). Intra-assay imprecision of 4.4% was calculated from duplicate analyses and expressed as the median of the range to the average of the duplicates. Inter-assay imprecision of <10% was determined using two quality control CSF pool samples.

### Ethical approval and patient consents

The study was approved by the Ethics Committee of the University of Tuebingen (26/2007BO1, 404/2010BO1, 199/2011BO1, 702/2013BO1 and 428/2018BO2). All participants gave written informed consent.

### Statistical analysis

Statistical analysis was performed using SPSS 26.0 software for Windows (IBM). Group comparisons of continuous data were analyzed using ANCOVA, including age and disease duration as co-variates.

Pearson’s correlation was used to evaluate associations between inflammatory markers with clinical data (H&Y, UPDRS-III, LEDD, MoCA, prevalence of cognitive impairment, interval until cognitive impairment) and with CSF markers for neurodegeneration (Aβ_1-42_, total-Tau, p181-Tau, NFL and total α-synuclein). Given that there are known sex differences in inflammation profile, correlation analyses were stratified by sex (Supplemental Table 2). Inflammatory markers with more than three standard deviations were excluded from the respective analyses. Only correlations with at least ten valid sample pairs and with a correlation coefficient >0.1 were considered meaningful (irrespective of the *p* values were <0.05).

Inflammatory markers (ICAM-1, Interleukin-8, MCP-1, MIP-1 beta, SCF and VEGF) showed a statistically significant and meaningful (correlation coefficient >0.1) association with clinical data and with neurodegeneration-associated CSF markers (Aβ_1-42_, total-Tau, p181-Tau, NFL and total α-synuclein) were analyzed by Kaplan–Meier survival curves and Cox regression. Inflammation group, age, sex and disease duration were modelled as interacting co-variate and evaluated by their impact on the longitudinal development of cognitive impairment. Analyses were done for PD patients with and without *GBA* mutations separately. PD_GBA_WT_ patients were divided into quartiles and PD_GBA_ patients into tertiles (due to limitation in sample size). As this study was exploratory, we did not correct for multiple testing.

### Reporting summary

Further information on research design is available in the Nature Research Reporting Summary linked to this article.

## Supplementary information

Supplementary Tables 1 and 2

Reporting Summary

## Data Availability

Anonymized data were available upon request to: kathrin.brockmann@uni-tuebingen.de.
