# Supplementary material for: Inflammatory CSF profiles and longitudinal development of cognitive decline in sporadic and GBA-associated PD
Source: NPJ Parkinsons Dis. 2023 Mar 11;9:38. doi: 10.1038/s41531-023-00476-2 (PMC10008539; doi:10.1038/s41531-023-00476-2)
Supplement: Supplementary file 1 — Supplementary Tables 1 and 2 [file 41531_2023_476_MOESM1_ESM.pdf]

Supplemental Table 1: Correlation between CSF inflammatory markers with demographics and clinical characteristics in Dementia with Lewy Bodies

	Age	Age at onset	Disease duration	H&Y (m n=41; f n=19)	UPDRS-III (m n=17; f n=4)	MoCA (m n=41; f n=18)	LEDD (m n=33; f n=15)
Eotaxin-1	n.s.	n.s.	n.s.	n.s.	n.s.	n.s.	n.s.
FactorVII	n.s.	n.s.	n.s.	n.s.	n.s.	n.s.	n.s.
ICAM-1	n.s.	n.s.	n.s.	n.s.	n.s.	n.s.	n.s.
Interleukin-1ra	n.s.	n.s.	n.s.	n.s.	n.s.	n.s.	n.s.
Interleukin-2	n.s.	n.s.	n.s.	n.s.	n.s.	t: 0.304* n=43	n.s.
Interleukin-4	n.s.	n.s.	n.s.	n.s.	n.s.	t: 0.359* n=42	n.s.
Interleukin-6	n.s.	n.s.	n.s.	n.s.	n.s.	n.s.	n.s.
Interleukin-7	n.s.	n.s.	n.s.	n.s.	n.s.	t: 0.482** n=35<br>f: 0.742** n=11	n.s.
Interleukin-8	n.s.	n.s.	n.s.	n.s.	n.s.	n.s.	n.s.
Interleukin-12p40	n.s.	n.s.	n.s.	n.s.	n.s.	n.s.	t: -0.353* n=38<br>m: -0.432* n=26
Interleukin-18	n.s.	n.s.	n.s.	t: -0.331* n=53<br>m: -0.353* n=35	n.s.	f: 0.515* n=17	n.s.
MCP-1	n.s.	n.s.	n.s.	n.s.	n.s.	f: 0.565* n=18	m: 0.368* n=33
MIP1-alpha	n.s.	n.s.	n.s.	n.s.	n.s.	n.s.	n.s.
MIP1-beta	n.s.	n.s.	n.s.	n.s.	m: -0.499* n=17	n.s.	n.s.
MMP3	n.s.	t: 0.255* n=66	n.s.	f: 0.491* n=19	n.s.	t: -0.275* n=59<br>f: -0.489* n=18	n.s.
MMP9	n.s.	n.s.	n.s.	n.s.	n.s.	n.s.	n.s.
SCF	t: 0.303* n=67	t: 0.319** n=66<br>f: 0.478* n=21	f: -0.492* n=21	n.s.	n.s.	n.s.	t: 0.313* n=48
TNF-alpha	n.s.	n.s.	n.s.	n.s.	n.s.	n.s.	n.s.
VEGF	n.s.	n.s.	n.s.	f: -0.536* n=19	n.s.	n.s.	m: 0.399* n=33

Pearson correlation: *p<0.05; **p<0.01; n.s. = not significant

t: total; m: males ; f: females

Supplemental Table 2: Baseline demographic and clinical data from people with Parkinson´s Disease stratified by GBA mutation and sex

	PD_{GBA_WT} male n=256	PD_{GBA_WT} female n=144	p-value PD_{GBA_WT}	PD_{GBA} male n=66	PD_{GBA} female n=32	p-value PD_{GBA}
Age, years	65 ± 10	66 ± 10	0.398	62 ± 10	65 ± 9	0.136
Age at onset, years	59 ± 11	59 ± 11	0.836	54 ± 11	56 ± 8	0.321
Disease duration, years	7 ± 5	7 ± 5	0.204	8 ± 5	9 ± 6	0.479
H&Y	2.1 ± 0.6	2.1 ± 0.6	0.849	2.2 ± 0.7	2.2 ± 0.7	0.990
UPDRS-III	27 ± 12	24 ± 11	0.015	27 ± 12	27 ± 11	0.961
MoCA	25 ± 4	25 ± 5	0.981	25 ± 4	24 ± 5	0.751
LEDD	576 ± 427	532 ± 490	0.356	695 ± 476	538 ± 328	0.101
Amyloid$\beta_{1\text{-}42}$ [pg/ml]	706 ± 264	717 ± 274	0.706	733 ± 267	653 ± 197	0.144
total-Tau [pg/ml]	224 ± 101	275 ± 158	<0.001	252 ± 150	231 ± 93	0.491
phospho181-Tau [pg/ml]	40 ± 16	45 ± 20	0.003	40 ± 17	39 ± 12	0.814
NFL [pg/ml]	1065 ± 1031	958 ± 1121	0.358	954 ± 625	800 ± 563	0.250
α-synuclein [pg/ml]	574 ± 254	709 ± 364	<0.001	550 ± 228	532 ± 308	0.744
Eotaxin-1	57.4 ± 7.5	56.4 ± 8.3	0.217	57.3 ± 7.5	58.7 ± 7.0	0.381
FactorVII	441 ± 191	429 ± 214	0.629	503 ± 224	423 ± 146	0.099
ICAM-1	516 ± 178	503 ± 184	0.491	517 ± 194	474 ± 154	0.269
Interleukin-1ra	19.5 ± 8.7	19.0 ± 8.5	0.637	20.7 ± 10.7	19.7 ± 9.2	0.698
Interleukin-2	10.6 ± 5.8	10.8 ± 5.6	0.838	11.3 ± 6.1	10.5 ± 7.6	0.656
Interleukin-4	8.08 ± 4.12	8.18 ± 3.88	0.861	9.18 ± 5.29	8.44 ± 4.53	0.559
Interleukin-6	1.01 ± 0.40	1.08 ± 1.12	0.483	1.07 ± 0.40	0.85 ± 0.27	0.035
Interleukin-7	4.38 ± 2.73	3.88 ± 2.10	0.151	4.87 ± 3.10	3.80 ± 2.26	0.207
Interleukin-8	40.8 ± 14.1	40.0 ± 26.1	0.707	42.0 ± 11.8	36.9 ± 11.6	0.050
Interleukin-12p40	0.12 ± 0.06	0.12 ± 0.05	0.930	0.12 ± 0.06	0.12 ± 0.06	0.679
Interleukin-18	8.61 ± 4.20	7.61 ± 3.32	0.039	9.12 ± 4.84	8.71 ± 3.63	0.712
MCP-1	707 ± 241	641 ± 211	0.006	719 ± 223	699 ± 205	0.669
MIP-1 alpha	9.99 ± 4.82	11.2 ± 4.9	0.158	10.1 ± 4.76	9.92 ± 4.94	0.919
MIP-1 beta	63.9 ± 19.6	64.2 ± 17.8	0.870	65.8 ± 19.7	64.5 ± 14.7	0.743
MMP3	158 ± 67	138 ± 59	0.004	142 ± 67	127 ± 70	0.310
MMP9	6681 ± 3468	7263 ± 3409	0.130	6474 ± 3105	6338 ± 3231	0.851
SCF	73.1 ± 22.5	77.6 ± 26.1	0.071	77.3 ± 30.5	69.9 ± 20.6	0.217
TNF-alpha	3.83 ± 1.47	3.52 ± 1.00	0.195	3.14 ± 0.93 n=33	3.14 ± 0.92 n=13	0.998
VEGF	35.9 ± 6.1	34.9 ± 6.2	0.132	37.6 ± 5.9	34.1 ± 5.5	0.005

Data are presented as mean and standard deviation. p-values were calculated by ANOVA
